# Neonatal Exhaled Breath Sampling for Infrared Spectroscopy: Biomarker Analysis

**DOI:** 10.1021/acsomega.4c02635

**Published:** 2024-07-02

**Authors:** Nadia Feddahi, Lea Hartmann, Ursula Felderhoff-Müser, Susmita Roy, Renée Lampe, Kiran Sankar Maiti

**Affiliations:** † Center for Translational and Neurobehavioural Sciences CTNBS, Department of Pediatrics I, Neonatology, University Hospital Essen, University of Duisburg-Essen, Hufelandstraße 55, Essen 45147, Germany; ‡ Research Unit of the Buhl-Strohmaier Foundation for Cerebral Palsy and Pediatric Neuroorthopaedics, Department of Orthopaedics and Sports Orthopaedics, TUM School of Medicine and Health, University Hospital Rechts der Isar, Technical University of Munich, Ismaninger Straße 22, 81675 Munich, Germany; § Markus Würth Professorship, Technical University of Munich, Ismaninger Straße 22, 81675 Munich, Germany; ∥ TUM School of Natural Sciences, Department of Chemistry, Technical University of Munich, 85748 Garching, Germany; ⊥ Max-Planck-Institut für Quantenoptik, Hans-Kopfermann-Straße 1, 85748 Garching, Germany

## Abstract

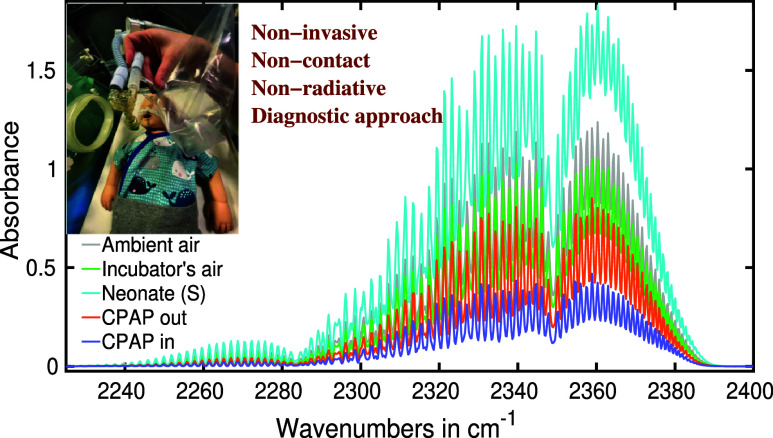

Monitoring health conditions in neonates for early therapeutic intervention in case deviations from physiological conditions is crucial for their long-term development. Due to their immaturity preterm born neonates are dependent on particularly careful physical and neurological diagnostic methods. Ideally, these should be noninvasive, noncontact, and radiation free. Infrared spectroscopy was used to analyze exhaled breath from 71 neonates with a special emphasis on preterm infants, as a noninvasive, noncontact, and radiation-free diagnostic tool. Passive sample collection was performed by skilled clinicians. Depending on the mode of respiratory support of infants, four different sampling procedures were adapted to collect exhaled breath. With the aid of appropriate reference samples, infrared spectroscopy has successfully demonstrated its effectiveness in the analysis of breath samples of neonates. The discernible increase in concentrations of carbon dioxide, carbon monoxide, and methane in collected samples compared to reference samples served as compelling evidence of the presence of exhaled breath. With regard to technical hurdles and sample analysis, samples collected from neonates without respiratory support proved to be more advantageous compared to those obtained from intubated infants and those with CPAP (continuous positive airway pressure). The main obstacle lies in the significant dilution of exhaled breath in the case of neonates receiving respiratory support. Metabolic analysis of breath samples holds promise for the development of noninvasive biomarker-based diagnostics for both preterm and sick neonates provided an adequate amount of breath is collected.

## Introduction

Health monitoring of preterm born, as well as term born neonates with complications such as perinatal asphyxia or sepsis, remains a major challenge for clinicians, despite recent improvements in survival rates due to advancements in obstetrics and neonatal intensive care.^1−3^ In preterms, challenges arise from numerous problems that may affect their developmental progress.^4−6^ In particular, clinicians face a significant challenge in the complex interplay between prenatal maternal health factors and potential immaturity complications such as bronchopulmonary dysplasia (BPD), inflammatory events (sepsis, necrotizing enterocolitis—NEC) or intraventricular hemorrhage (IVH), and socioeconomic factors.^7−9^ The risk for cerebral palsy (CP) or other neurological disorders for example, inversely correlates with gestational age (GA); meaning that the earlier children are born, the higher the risk for neurological impairment.^10,11^ Despite the use of sophisticated methods for monitoring health and diagnostic scores, it is almost impossible to predict the clinical or neurological outcomes of these children during their perinatal inpatient stay. For example, although, the risk of infantile CP is related to GA, usually it is diagnosed at the age of 1–2 years.^12−14^ This is because children can be in a transitional phase during their development, in which it is not yet clear whether permanent disability will develop or whether the developmental delay will normalize.^15,16^ Therefore, monitoring the health status of these neonates is crucial to effectively address arising problems potentially affecting their development.^17−19^ In addition, it is important to make an early developmental prognosis, both to initiate supportive interventions early and to communicate this prognosis to parents.^20,21^ To overcome these challenges, the ideal health monitoring approach would be noninvasive, noncontact, and radiation-free diagnostic methods to avoid unnecessary stress, risks, and side effects for the children.

In this regard, metabolites-based diagnosis certainly would be an attractive option,^22,23^ since many metabolites can be collected noninvasively via, urine, faeces, exhaled breath, etc.^24,25^ It is an established fact that metabolites, which are byproducts of biochemical reactions in the living cell, carry specific cellular information.^26,27^ Analyzing the chemical compositions of these metabolites allows insights into the body’s internal chemistry, facilitating better monitoring of the body’s state,^22,28^ as well as an indication of diseases that remain asymptomatic in their initial stages.^29−31^ As bioprobe, exhaled breath stands out as a particularly promising source of metabolites for clinical diagnosis,^32^ primarily due to its noninvasiveness, patient-friendly nature, and rapid processing capabilities. Since the 1970s, numerous studies have investigated breath-based metabolites in the adult population.^33^ The primary reason for selecting adults as the test group is the convenience of sample collection. Currently, individuals being tested still need to exhale directly into the measurement system or a storage device. However, there remains significant debate surrounding sample collection, even for adults.^34^ Certain demographics, such as infants or individuals who are immobile, weak, or in intensive care, face challenges in performing the required exhalation maneuvers for breath analysis. Recent research has made strides in addressing these challenges. In one study, breath samples were collected from 16 infants using Nalophan bags.^35^ In another study, the exhaled breath of neonates was directly collected from the respiratory support system using a sampling pump.^36^ Furthermore, beyond newborns, research has expanded to include the analysis of volatile organic compounds (VOCs) in the exhaled breath of pregnant sheep. This exploration holds significant promise for identifying pregnancies complicated by intra-amniotic infections.^37^

Various experimental techniques, e.g., different mass spectrometry (MS) techniques, infrared (IR) spectroscopy, electronic nose (e-nose), etc. are rapidly developing to reveal gaseous metabolites and have already demonstrated their contributions to breath research.^38,39^ It is worth noting that MS techniques currently play the most significant role in breath research, identifying hundreds of VOCs with an impressive sensitivity down to 100 ppt (parts per trillion).^40^ However, the complex and underdeveloped sample preparation process in MS leads to accuracy issues, which raises doubts about its reliability for medical diagnosis.^41^ Furthermore, MS devices are costly and bulky in size. In contrast, e-nose devices are more cost-effective and compact.^42,43^ They use an array of chemical sensors to mimic the human olfactory system.^44^ However, their “black box” nature has resulted in varying outcomes across research groups. Moreover, e-nose devices are not suitable for the identification of metabolites.^45^

Compared to mass spectrometry and e-nose devices, infrared spectroscopy offers several advantages in the identification and quantification of molecular compositions from a mixture of molecules in the gas. It uses the most fundamental molecular properties, e.g., molecular vibrations, as a probe to identify the molecule through structural analysis.^46−49^ Infrared light is used to stimulate molecular bonds and consequently, the absorption of light during their vibrational motion is recorded by an infrared detector. This process leads to the generation of a distinctive set of spectral features for each molecule in the acquired infrared spectra. In the realm of clinical spectroscopy, these distinct spectral features are commonly referred to as “fingerprints” of the molecule.^50,51^ The precise characteristics, including the position, intensity, and morphology of these molecular fingerprints, play a crucial role in advancing metabolite-based infrared diagnostics.^52,53^

Notably, infrared spectroscopy has already demonstrated its efficacy in identifying biomarkers for various diseases in the adult population.^14,29^ Here, we hypothesize that infrared spectroscopy for breath biomarker analysis can also contribute to neonatal health monitoring, provided a feasible means of collecting an adequate quantity of exhaled breath can be established. We outline a procedure for the collection of neonatal exhaled breath and the corresponding measurement techniques.

## Experimental Method

### Sample Collection

**Figure 1:**
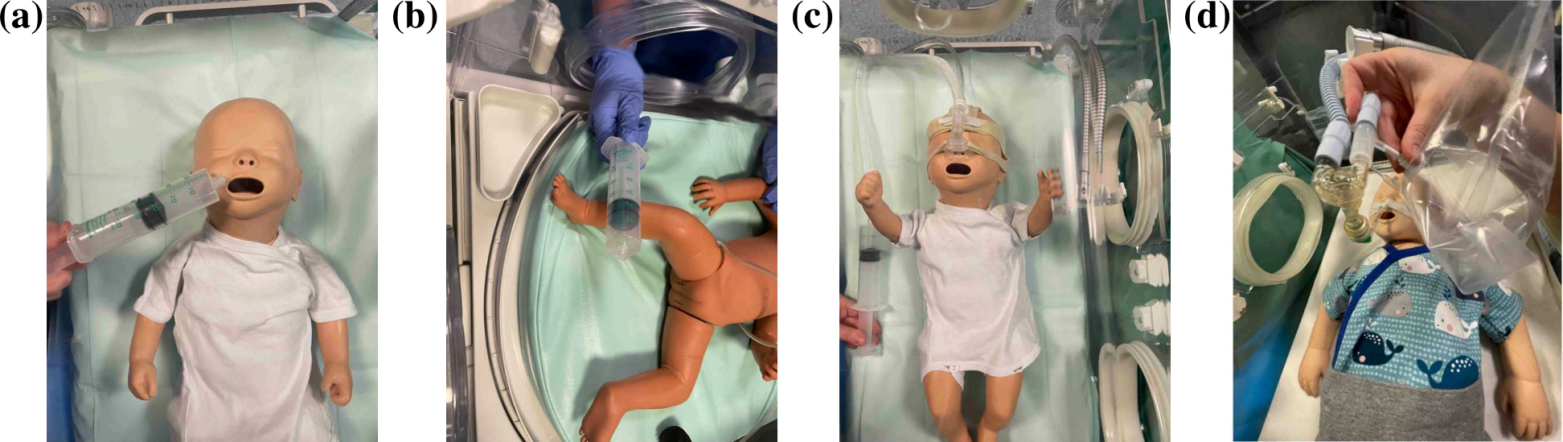
Collection procedures of exhaled breath samples from neonates are depicted with the help of a model. Passive breath sample collection for neonates under various conditions, including (a) spontaneously breathing neonate, (b) air from the incubator with neonate, (c) neonate with CPAP delivered via ”infant flow” as respiratory support, and (d) intubated neonate.

Preterms or critically ill newborn infants are kept in a meticulously controlled environment, akin to a pristine clean room. The use of any equipment is strictly limited. Therefore, all spectroscopic measurements were conducted offline. The current state-of-the-art method for breath sample collection and storage for infrared diagnosis involves using a single-use Tedlar bag (Supelco Tedlar Bags, LOT#: 10311LC19C).^54^ The sample collection bags are equipped with a valve through which a participant can actively blow exhaled air, just as simple as blowing a balloon. In the case of newborns, sample collection needed to be performed passively, representing the major challenge for the development of this noninvasive, noncontact, metabolites-based diagnostic tool. Further adjustments were required depending on the type of respiratory support (invasive, noninvasive CPAP) and also depending on the type of care (incubator, baby cod). The collection procedures are presented in Figure 1 using a model.

### Study Design

This prospective cohort single-center study was approved by the Ethics Committee of the Faculty of Medicine at the Technical University of Munich (Reference Number: 146/21 S-EB) and the Ethics Committee of the Medical Faculty of the University of Duisburg-Essen (Reference Number: 21-10068-BO), and conducted in accordance with the Declaration of Helsinki. All experimental protocols, as well as procedures related to patients’ data privacy and personal interests, received approval. Before collecting breath samples, the parents of the neonates were duly informed about the study and provided their written consent.

**Figure 2:**
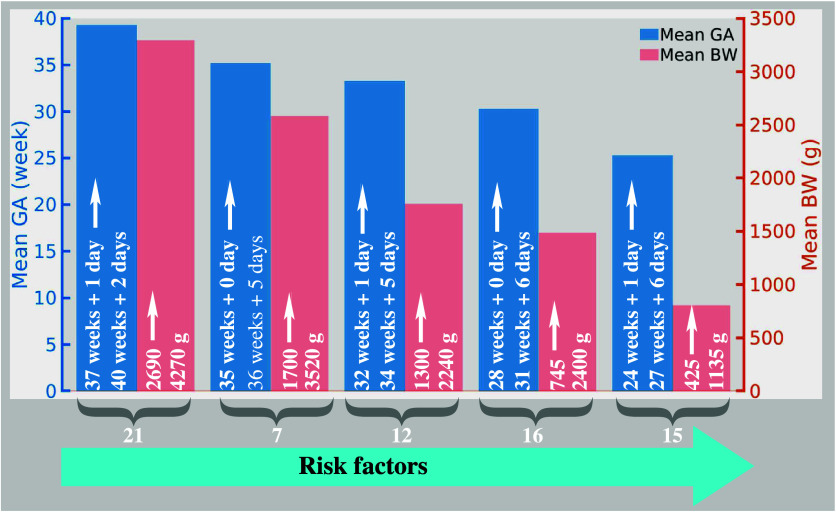
Sample population according to the GA of neonates. A blue color bar plot illustrates the mean GA for each group, with the lowest and highest GAs noted on the bars. Similarly, the red bars depict the lowest, highest, and mean BW. The left side scale corresponds to the mean GA, while the right side scale corresponds to the mean BW.

Following parental informed consent 50 preterm and 21 healthy term born at the University Hospital Essen between 01/04/2022 and 24/01/2023 with gestational age (GA) between 24_+1_ (the subscript stands for days) and 40_+2_ weeks (mean GA 32_+6_ weeks) and birth weight (BW) between 425 and 4270 g (mean BW 1985.6 g) were included in this study (Figure 2). The total number of samples was subdivided by GA into five subgroups. Neonates born at or beyond 37_+0_ weeks were categorized as “term born”. Breath samples of 21 healthy term born infants with an average GA of 39_+3_ weeks (born between 37_+1_ and 40_+2_ weeks) and an average BW of 3294 g (born with a BW between 2690 and 4270 g) were collected. The second group consisted of ”late preterm” neonates born between 35_+0_ and 36_+6_ weeks (*n* = 7, mean GA 35_+2_ (35_+0_ to 36_+5_)) and mean BW of 2585 g (ranging from 1700 to 3520 g). The “early preterm” group included infants born between 32_+0_ and 34_+6_ weeks (*n* = 12, mean GA 33_+3_ (32_+1_ to 34_+5_)) and mean BW of 1756 g (ranging from 1300 to 2240 g). The subsequent group was “very preterm”, comprising neonates born between 28_+0_ and 31_+6_ weeks (*n* = 16, mean GA 30_+3_ (28_+0_ to 31_+6_)) mean BW 1487 g (ranging from 745 to 2400 g). In the “extreme preterm” category we considered infants born <28_+0_ weeks (*n* = 15 mean GA of 25_+3_ (24_+1_ to 27_+6_), mean BW of 806 g (ranging from 425 to 1135 g).

#### Neonates Breathing Spontaneously (S)

Irrespective of the GA, the majority of the neonates in the study group breathed spontaneously. For them breath sampling was performed with a 50 *ml* syringe (Original Perfusor Syringe 50 *ml*, LOT: 21E18D8004) held as near as possible between mouth and nose, without touching the neonate’s skin, and slowly aspirating the exhaled air (see Figure 1a). The collected breath sample was injected into the Tedlar bag via a needle through the intended valve. This procedure was repeated 20 times to collect about 1 Liter of sample from each neonate. An equivalent volume of ambient air was collected in a separate Tedlar bag to serve as a point of comparison (reference air) for the samples collected from spontaneous breathing.

#### Neonates Requiring Incubator Care

For small preterm born neonates incubator care is essential due to their immature thermoregulation system.^55^ To investigate the influence of exhaled breath in the entire incubator’s air, a few samples (reference sample) were collected far from the infant’s nose (see Figure 1b). Otherwise, samples were collected close to the nose. All collections were performed using a 50 mL syringe and followed the same procedure as described above in (a).

#### Neonates on CPAP (Infant Flow) as Respiratory Support (CPAP)

Continuous Positive Airway Pressure (CPAP) is a method of applying positive airway pressure by continuously delivering air into the respiratory tract.^56^ This technique is employed to ensure a consistent pressure level, thereby ensuring the continual patency of the airway in individuals who are naturally breathing (S). Samples were collected directly from the exhalation tube of the system by holding a 50 mL syringe near the tube opening and slowly aspirating the air (see Figure 1c). Subsequently, the sample collection procedure was followed as explained above. Collected air from the inhalation tube served as a reference sample for neonates on CPAP.

#### Neonates on Invasive Ventilation (Draeger Babylog VN500, IT)

Invasive ventilation involves the use of a ventilator to deliver air into the airways through an endotracheal tube, maintaining a specific pressure level.^57^ In the study period, only one neonate required invasive respiratory support. The sample was collected by directly connecting the Tedlar bag over an interponate to the closed system of the respirator (see Figure 1d).

**Figure 3:**
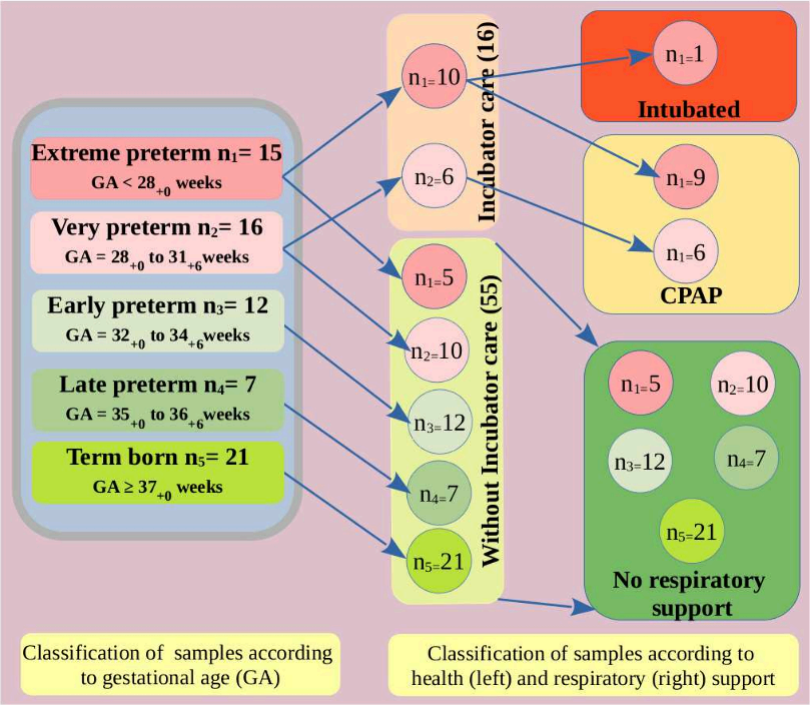
Classification of collected samples according to the GA of the neonates as well as their life and respiratory support. The subscript in GA indicates the days, e.g., GA = 36_+6_ weeks mean 36 weeks and 6 days.

All collected samples were classified according to the GA and type of respiratory support illustrated in Figure 3. It is important to note that a single syringe was used for each neonate, to prevent sample contamination.

### Sample Preparation

All samples collected at University Hospital Essen were stored at 4 °C for a maximum of 7 days prior to transfer to the Department of Physics, Ludwig-Maximilians University of Munich for spectroscopic analysis. Samples were transported in well-sealed polystyrene boxes with cold packs. Samples arrived within 24 h and were measured immediately upon arrival. It is important to note that the majority of samples were transferred within 2–3 days of collection, therefore, they were analyzed 2–4 days after collection. In such a short time frame, we do not anticipate any leakage or degradation of the samples. This was confirmed by a separate study involving 10 aliquoted samples from healthy adults. All samples were stored at 4 °C and analyzed up to 30 days after collection. There was no significant change in the molecular concentration of breath metabolites observed until day 20. However, a slight decrease (approximately 20% by the 30^th^ day) in CO_2_ concentration was noted thereafter. Notably, there were practically no deviations observed for the other molecules analyzed, which are larger in size than CO_2_.

**Figure 4:**
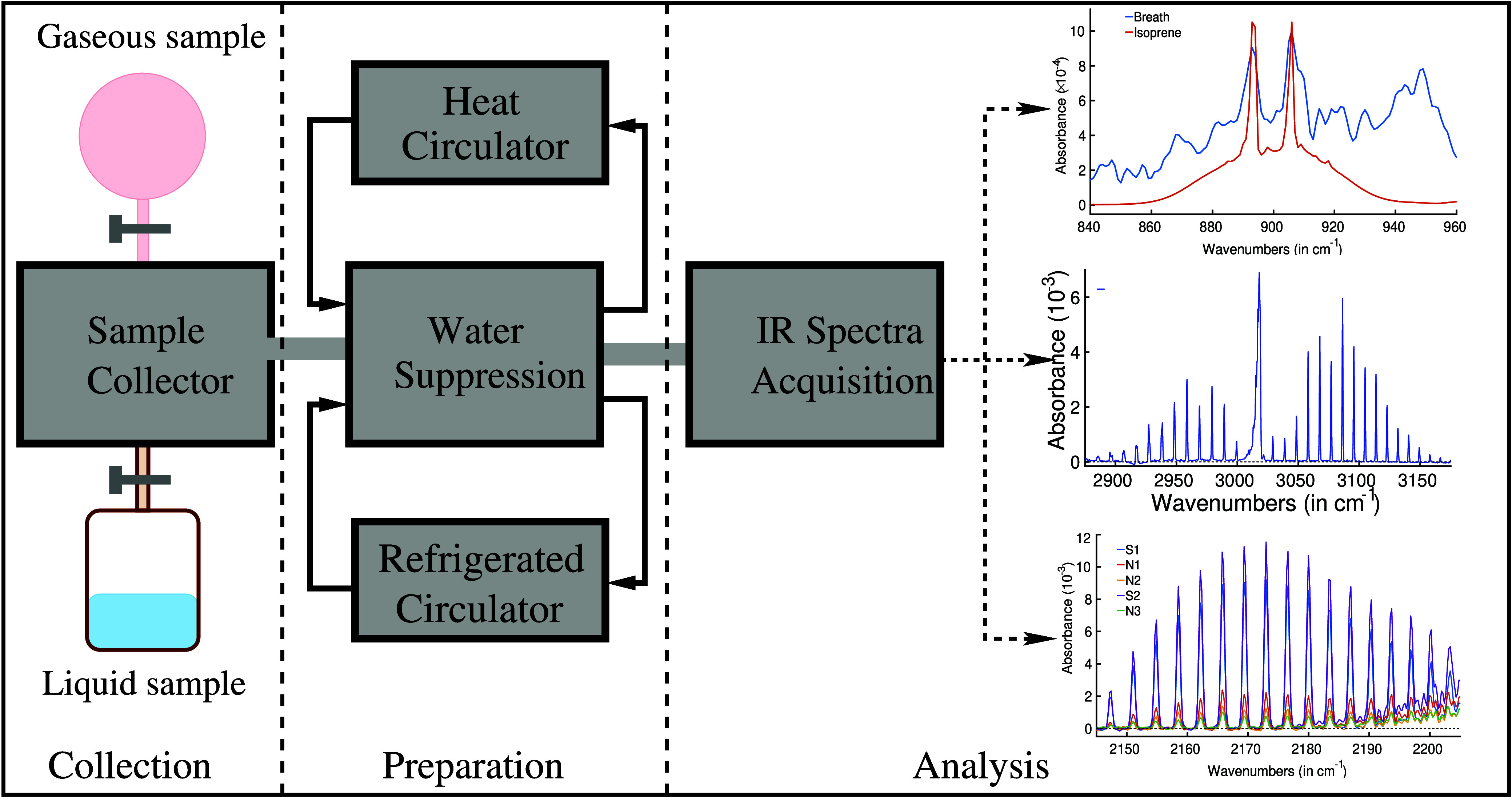
Diagram of the experimental scheme for gaseous biofluid analysis by infrared spectroscopy. It consists of three major parts: (1) Collection—in this part, breath or headspace of liquid biofluids is collected; (2) Preparation—a water-suppressed sample is prepared for infrared spectroscopy when gaseous biofluids are passing through the “Water Condenser”; and (3) Analysis—water suppressed gaseous sample is collected in a multipass gas cell and measured with an FTIR spectrometer.

Compared to the other breath sample analysis techniques, infrared spectroscopy requires a minimal sample preparation process. The primary challenge encountered when applying infrared spectroscopy to exhaled breath is the substantial presence of water within the samples. However, a recent advancement in water suppression technique for gaseous biofluids has introduced a promising avenue for conducting infrared spectroscopic analyses on gaseous biofluids.^58^ To prepare samples amenable to infrared spectroscopic analysis, a self-built water suppression system was employed. Figure 4 provides a schematic overview of the water suppression technique, coupled with the spectroscopic measurement unit. The details of the system and its working principle were reported earlier.^58^ In brief, the sample preparation technique consisted of two major units, namely, (1) a sample collector and (2) a sample preparation unit. (1) The sample collector system was designed in such a way that it could accept gaseous samples as well as the headspace of liquid biofluids. Prior to sample injection into the sample collector, the entire sample path was evacuated (down to a pressure level of 10^–5^ mbar) using two vacuum pumps. This process effectively eliminates any residual contamination from previous measurements. Breath samples were transferred to the empty sample collector by releasing the valve. (2) The sample preparation unit was composed of essential components, including a water condenser and both heat and refrigerated circulators. The water condenser was designed as a sealed metal chamber housing a 12-m-long copper tube coiled into a spiral configuration. This copper tube served as the conduit for transferring the breath sample from the sample collector to a measurement cell. Prior to its passage through the water condenser, the chamber was meticulously cooled to a temperature of −60 °C using a refrigerated circulator. Once the water condenser reached this frigid temperature, the breath sample was allowed to flow through the spiral copper tube at a precisely controlled rate of 3 mL per second. During this transit through the cold copper tubing, a substantial amount of water vapor was effectively removed from the sample. Remarkably, an impressive water vapor reduction factor exceeding 2500 was achieved when the sample passed through the water condenser at −60 °C. Subsequently, the water-suppressed gas-phase biofluid was transferred to the multipass sample cell. Following each experimental run, the copper tube undertook a cleaning procedure by heating up the chamber to 45 °C with the heat circulator and vacuum pumps.

### Spectroscopic Measurements

All the spectroscopic measurements of breath samples were performed using an FTIR spectrometer (Vertex 70, Bruker Optics GmbH, Germany). The spectrometer operated within a spectral range of 500–4000 cm^–1^ and utilized a 4-m optical path length along with a 2-L “White cell” (Bruker Optics GmbH, Germany) to hold gaseous samples for spectroscopic analysis. The absorption spectra of breath samples were captured by a liquid nitrogen-cooled MCT detector. For all measurements, a 0.5 cm^–1^ spectral resolution was used. To reduce the noise, 100 spectra were collected and averaged for each sample. The spectrometer demonstrated a sensitivity of 10 parts per billion (ppb) for VOCs within the range of moderate water absorption.

### Spectroscopic Data Analysis

The absorption spectra of breath samples were analyzed by component analysis.^59,60^ Initially, significant spectral features were searched from the infrared spectra of breath. Gas-phase molecular spectra were fitted with the observed spectral features in the breath sample using least-squares fitting in order to find out the best agreement.^59^ Usually, gas-phase molecular spectra were collected from commercial databases (e.g., PNNL,^61^ HITRAN,^62^ NIST^63^) or acquiring spectra experimentally as well as theoretically by quantum chemistry calculations.^59,64^

## Results and Discussions

The primary goal of our study was to establish the reliability of sample collection from neonates from different gestational age groups. It is a well-known fact that human exhaled air volume strongly depends on body weight.^65^ Therefore, a high dynamic range of exhaled air is expected from different sample groups as the body weight of the neonates largely varies. While we analyzed individually the collected exhaled breath samples of 71 neonates, for demonstration purposes, only one case from each group is presented graphically. The summary of our results is presented in Table 1.

**Table 1:** Number of Samples for Each Sampling Method in Which Metabolites Are Detected^a^

sampling methods		metabolites					
		carbon dioxide		carbon monoxide		methane	modified
		detected	elevated	detected	elevated		methane
spontaneous		55	55	55	47	55	15
incubator	CPAP	15	15	15	11	15	2
	Infantflow	1	1	1	1	1	1

a The elevation of absorption strength of metabolites was analyzed with respect to the corresponding reference spectra.

### IR Spectra of Room Air

To validate the reliability of our exhaled breath sampling methods, we initiated a comparative study involving the analysis of infrared spectra from four distinct sources: the ambient air, exhaled breath of neonates without respiratory support (Figure 1a), samples from the exhalation tube (CPAP, invasive ventilation) (Figure 1 c and d), and air collected from incubators with neonate inside (Figure 1b). The primary challenge we encountered was assessing the variability within these four sample types. To address this, we initially concentrated on examining the ambient air. The reason behind this is obvious. Since our specific target group was neonates, we collected samples in close proximity to their nose and mouth, ensuring no physical contact and minimizing the risk of contamination. Consequently, a substantial amount of ambient air was inadvertently mixed with the breath samples. To conduct an accurate analysis of these mixed samples, it became imperative to segregate the contribution of ambient air. Therefore, acquiring a comprehensive understanding of the characteristics of ambient air became an essential step in the study.

**Figure 5:**
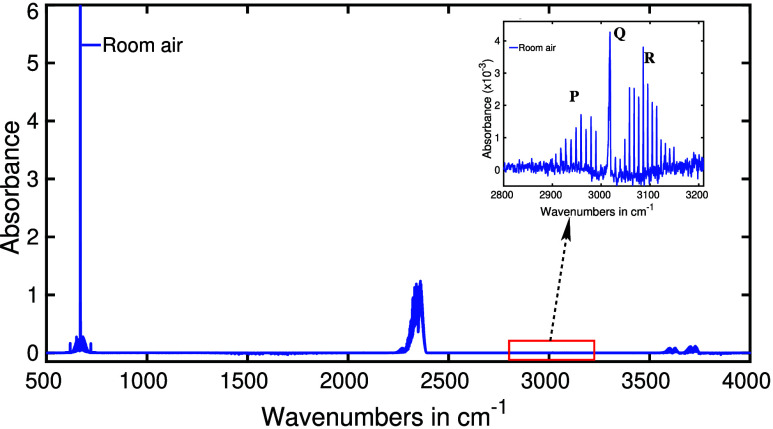
Infrared absorption spectra of room air. Inset is the 10^4^ times magnification of the same spectra at around 3000 cm^–1^.

A representative infrared spectrum of water-suppressed ambient air is depicted in Figure 5, revealing three prominent absorption peaks centered at approximately 670, 2350, and 3600 cm^–1^. These absorption peaks correspond to distinct vibrational modes of carbon dioxide (CO_2_).^62^ CO_2_ is a prevalent component of atmospheric air, existing at a relatively high concentration of around 400 ppm (parts per million).^66^ Furthermore, humans also emit endogenous CO_2_ when exhaling, contributing to the atmospheric CO_2_ content. Fortunately, separating these two sources of CO_2_ is a straightforward task through digital subtraction techniques.^59^ However, extracting specific biochemical information in the body by CO_2_ is rather challenging, since a majority of biochemical processes in the human body produce CO_2_. While CO_2_ may not be highly informative for health monitoring, its significance in our study is elucidated in the subsequent section.

The initially unremarkable spectra became significant when spectra were zoomed along the “absorbance axis.” This amplification, spanning three to four orders of magnitude, unveils numerous molecular signatures that provide a deeper insight into the body’s state. To illustrate, a three-order magnification centered around the spectral position at 3000 cm^–1^ exposes a distinct spectral pattern attributed to methane, comprising its well-defined **P**, **Q**, and **R** branches^58^ (visible in the inset of Figure 5). Additionally, several other molecular signatures were detected, and their explanations were described in subsequent sections.

### Identification of Breath of Neonates

The next question is whether passively collected breath samples obtained from different groups of neonates contained sufficient information for health monitoring through infrared spectroscopy. “Passively collected” means that these samples consisted of a combination of exhaled breath and ambient air.

To address this question, a comprehensive study was performed using samples from various groups. It is well-established that many biochemical reactions within cells generate CO_2_ as a byproduct. In general, CO_2_ is produced when carbohydrates and fats are metabolized.^67,68^ The cardiovascular system plays a crucial role in transporting CO_2_ from tissues to the alveolar membrane of the lungs. Due to the concentration gradient of CO_2_ between the alveolar air and the blood vessels in the alveolar membrane, leading to the diffusion of CO2 into the alveolar air. Consequently, exhaled breath contains a significantly higher concentration of CO2 compared to inhaled air. The variability of these samples was assessed by measuring CO_2_ levels, which serve as a reliable indicator.^69^ Given that endogenous CO_2_ significantly contributes to the composition of breath metabolites, the amount of CO_2_ content above the ambient (reference) CO_2_ level served as a practical measure of the proportion of exhaled breath in the collected sample.

**Figure 6:**
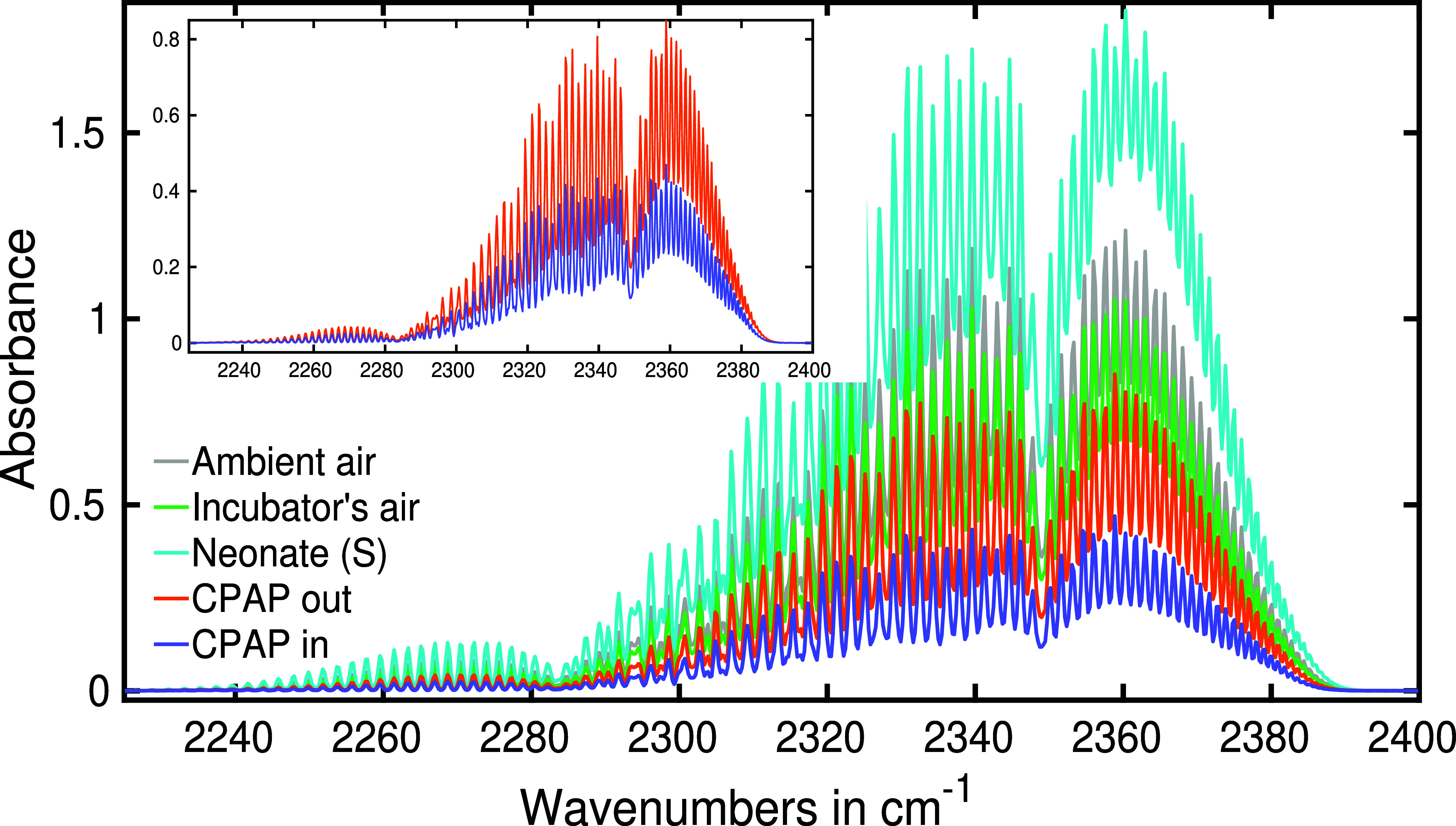
Infrared absorption spectra of ambient air, incubator’s air (with neonate), and exhaled air of a neonate with spontaneous (S) respiration, inlet and outlet air of a CPAP system. Spectra are zoomed around the absorption spectra of carbon dioxide.

In our practical analysis, we compared the strength of CO_2_ absorption in various sample types. Typically, the most pronounced CO_2_ infrared absorption peak is observed at around 2350 cm^–1^. Figure 6 demonstrates the zoomed-in spectra depicting the characteristics of CO_2_ absorption in different sample types including ambient air, air from an incubator (with an infant present), a sample from the inlet and outlet of CPAP and a sample of exhaled air collected from a baby without respiratory support.

It is noteworthy that the reference air varies depending on the respiratory support provided to neonates. In the simplest scenario, room air is considered as the reference for neonates without respiratory assistance. In Figure 6, the gray plot illustrates the reference spectra for nonrespiratory supported neonates. Incubators typically equipped with a controlled oxygen supply, result in lower CO_2_ levels compared to room air. To establish the reference for neonates in an incubator, we collected air samples distant from their mouths (refer to Figure 1b), represented by the green spectrum. As anticipated, the CO_2_ absorption strength was lower than the room air. For neonates on respiratory support, inhaled air is precisely controlled with elevated oxygen levels, and the inlet air served as the reference for these cases. This reference sample exhibits significantly lower CO_2_ compared to other references, as depicted by the blue line in Figure 6. These diverse reference air samples showcase distinct CO_2_ concentrations, forming the basis for further analysis.

Notably, samples collected near the neonate’s mouth exhibited the strongest CO_2_ absorption spectra. The CO_2_ absorption strength for this sample is 80% stronger than the corresponding reference, attributed to the neonate’s exhaled breath. To ensure the consistency of the sample collection process, we collected five consecutive samples from a single neonate, and the CO_2_ absorption strength remained consistent in each sample, indicating uniform and replicable collections.

The inset of Figure 6 displays the infrared spectra of the inlet and outlet air of CPAP. A more than 80% increase in CO_2_ levels in the outlet confirms the presence of neonate exhaled air. This affirms the capability of our sampling method and detection technique in accurately discerning neonate breath samples.

To estimate the minimum distance from mouth for effective sample collection, we conducted a dilution series with a healthy adult volunteer. An exponential drop in absorption strength with the distance was observed. Notably, when the sample was collected 10 cm distant from the nose, the CO_2_ absorption strength decreased by 50% compared to the collection closer to the nose without touching the skin. Here it is noted that samples were collected in the exhaled air flow direction.

### Carbon Monoxide

To additionally support our investigations on collected exhaled breath from neonates, we explored the absorption spectra of carbon monoxide (CO). Typically, carbon monoxide is present in the atmosphere at an extremely low concentration, measuring less than 100 ppb.^70^ This concentration varies based on factors such as population density, civilization, industrial activity, and geographical location. However, in the exhaled breath of healthy adults, a significant amount of CO exists as an endogenous metabolite produced due to oxidative stress in the lungs and inflammatory tissue injury.^71^

**Figure 7:**
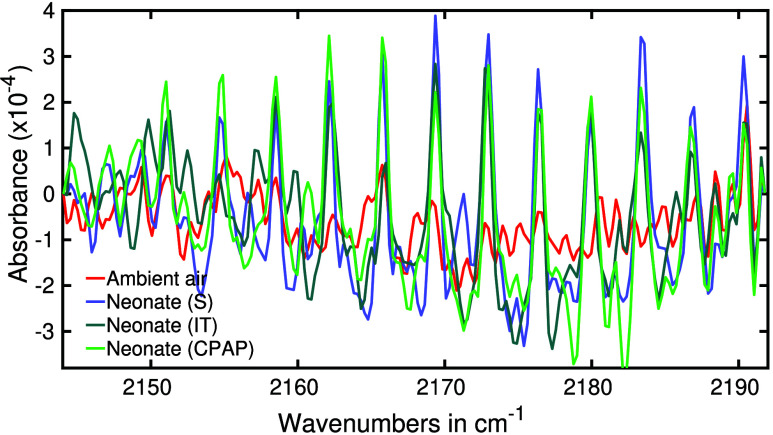
Infrared absorption spectra of ambient air along with exhaled breath from neonates with spontaneous (S) respiration, intubated (IT) and CPAP as respiratory support. The spectra are magnified around the absorption feature of carbon monoxide.

In the realm of infrared spectroscopy, the characteristic absorption spectrum of CO is typically located around 2170 cm^–1^. In human breath infrared spectra, the CO absorption feature is often obscured by the overlapping water absorption spectra. However, in our experiment, the robust water suppression techniques allowed us to clearly visualize the absorption spectra of CO. We have presented the absorption spectra of various sample types at CO absorption spectral region in Figure 7. In case of ambient air (red line), no measurable CO was observed. However, prominent spectral features of CO were observed for exhaled breath from neonates breathing spontaneously (S), intubated and invasively ventilated (IT) neonates, and neonates on noninvasive ventilation (CPAP) as respiratory support. This finding provides additional supportive evidence for the efficacy of our sampling method.

### Methane

Methane is one of the greenhouse gases present in the atmosphere. During inhalation, each individual inhales methane from ambient air, and a similar amount is expected in exhaled breath. In general, methane concentrations tend to be notably higher in a subset of the adult population, affirming its endogenous origin.^72^ This increased methane concentration in exhaled breath has been attributed to the presence of methanogenic bacteria,^73^ and it can vary among individuals based on factors such as ethnic background, diet, gut bacterial flora, and intestinal transit time.^74^

**Figure 8:**
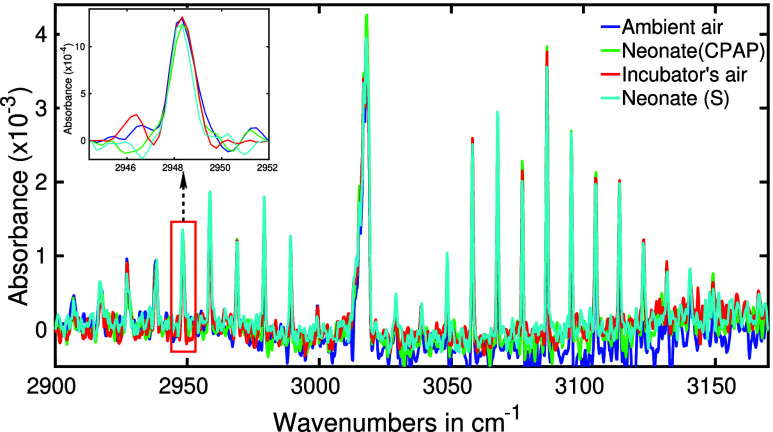
Infrared absorption spectra of ambient air, the sample from a neonate with CPAP respiratory support, air from an incubator, and exhaled air of a neonate with spontaneous (S) respiration. Spectra are zoomed around the absorption feature of methane.

To the best of our knowledge, there is currently no research available on methane levels in the exhaled breath of neonates. Therefore, this study represents the inaugural investigation into methane concentrations in the exhaled breath of neonates. In Figure 8, we present infrared spectra of methane obtained from the ambient air, exhaled air of a neonate (see Figure 1a), air from the outlet of infantflow tube (see Figure 1c), and the air from the incubator with neonate (see Figure 1b). It is important to note that the methane molecules were not isolated from the sample. Instead, the absorption spectral region of methane was magnified and depicted in the figure. Notably, all of these spectra exhibit striking similarities in terms of absorption strength. To provide a more detailed view of the spectral strength, we have zoomed in on the spectral peak at 2948 cm^–1^, which is shown as an inset in Figure 8. It is evident that the spectral peaks from all four samples perfectly overlap, indicating the absence of endogenous methane in the exhaled breath of these two particular neonates. However, it is worth noting that a minor increase up to 20% in methane absorption strength was observed in a couple of neonates within our study group. Determining the source of this slight elevation in methane absorption spectra remains a challenge. In general, it is observed that methane concentrations can increase 2–10 times in the adult population.^75^

**Figure 9:**
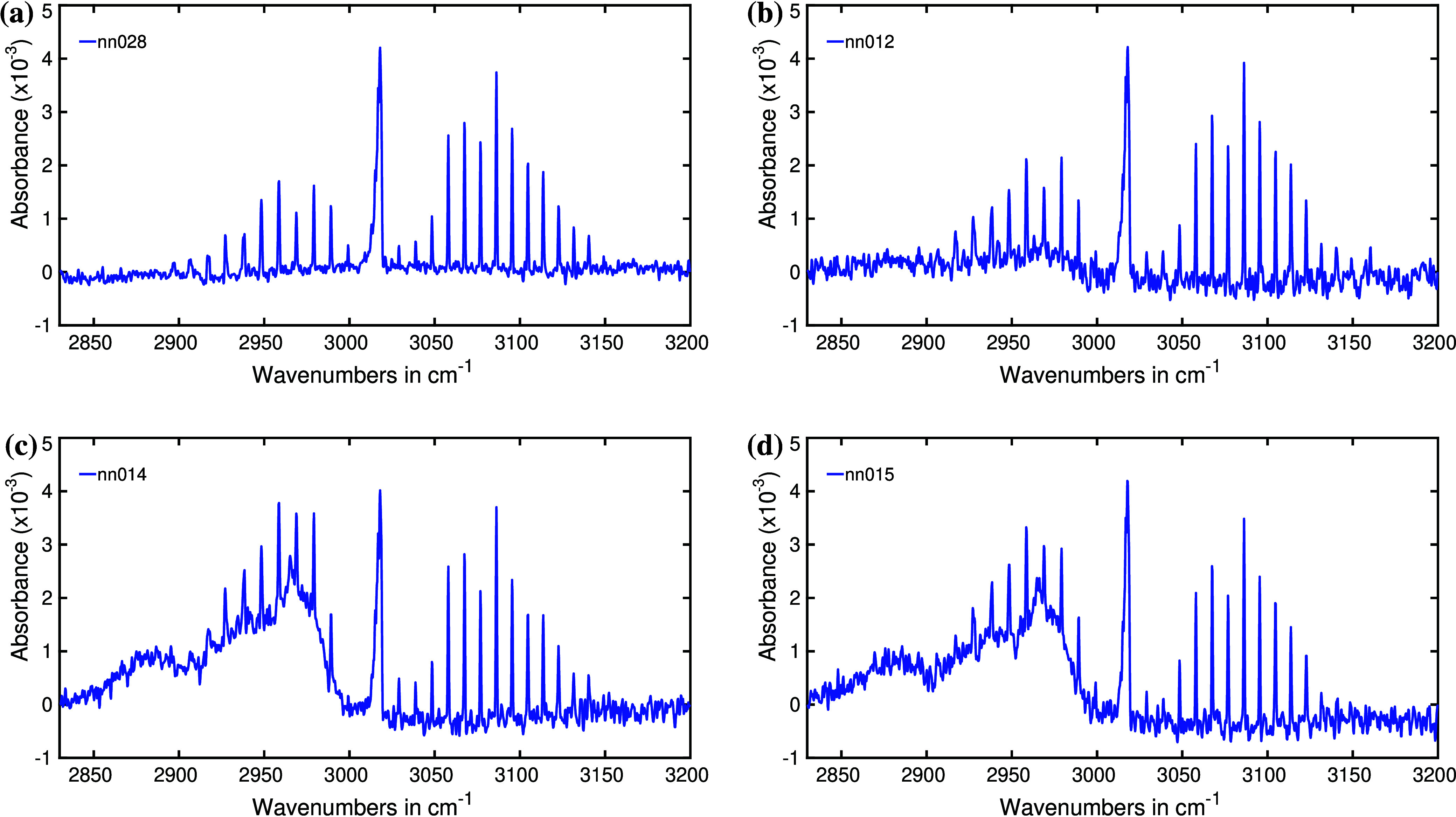
(a–d) Infrared spectra of breath in the spectral region of methane for four different neonates. The spectral feature of methane is modified due to the presence of unknown endogenous metabolites.

Although a minor variation in methane concentration is noted in 19 out of 71 neonates, it is insufficient to draw any meaningful conclusions regarding the gut bacterial flora. Instead, we observe a significant modification in the spectral characteristics of methane from a few neonates. For instance, Figure 9 displays spectra around 3000 cm^–1^ for exhaled breath from four individual infants. In Figure 9a, the spectra’s baseline appears largely uniform, as expected. When there is no overlap with other molecular spectra, methane spectra typically exhibit a flat baseline. However, in Figure 9b, a slight modification is detected in the **P**-branch of the methane spectra. A previous study documented a similar change in methane spectra.^76^ Nevertheless, in the current investigation, we do not have the opportunity to further explore the underlying causes of this spectral modification.

A similar modification is observed in both Figure 9c and Figure 9d, but its impact is significantly more pronounced than in Figure 9b. Interestingly, these two samples originated from twins. Regardless of the underlying reason for this spectral characteristic, both infants exhibit the same concentration of this specific metabolite. A similar spectral feature was observed in exhaled breath of 18 out of the 71 neonates. Notably, the spectral intensity of this attribute varies among individuals, suggesting that all 18 neonates possess this specific metabolite, although in varying concentrations.

Table 1 provides an overview of three metabolites discussed in this article. While we have identified many more metabolic signatures in the exhaled breath of neonates, methane, CO_2_, and CO are consistently present in all samples. However, the elevation of absorption strength is not uniform. In particular, no elevation of carbon monoxide was observed for a few samples, both in the case of spontaneous breathing and CPAP respiratory support. It is possible that those neonates exhaled significantly small amounts of carbon monoxide, falling below our detection limit. Another possibility is the nonuniformity in the sample collection. Despite the efforts of two skilled clinicians, the challenging health conditions of neonates may have affected the consistency of sample collection. This underscores the necessity to reassess our sample collection procedure to ensure uniformity, and we are actively working on refining the technique for this purpose. Furthermore, the analysis technique and identification of breath biomarkers can be further improved by employing GC-MS analysis in conjunction with the present FTIR technique.

## Conclusions

To the best of our knowledge, we demonstrate the first study on exhaled breath of 71 preterm and term born newborns using infrared spectroscopy. Four different methods were explored for collecting breath samples, depending on the type of respiratory support required.

Among the four sampling techniques investigated, the method involving sample collection between the nose and mouth was the simplest way of sample collection which yielded the most promising results. Exhaled breath and reference sample collections from neonates with respiratory supports seem more challenging compared to spontaneously breathing neonates. It is imperative to reassess and refine the sampling procedure.

By utilizing the infrared molecular spectral characteristics of carbon dioxide and carbon monoxide, the study demonstrated the effectiveness of the sample collection techniques in gathering exhaled breath from neonates. Although a slight increase in methane concentration was observed in the exhaled breath of 19 neonates, it is not enough to conclude its endogenous origin. However, the modification of methane spectra in 18 out of 71 neonates unambiguously manifests the exhaled breath sample collection. The exact cause of this spectral modification remains unidentified. Nevertheless, it is intriguing to note that this feature was consistently present in the case of twins, suggesting a potential shared physiological characteristic among them.

The developed breath collection method would definitely be extremely valuable for monitoring the health of newborns. Consequently, we believe this technique will empower us to identify biomarkers, with a particular focus on those associated with neurological diseases that may remain asymptomatic at a very early age.
